# Disease–treatment interactions in the management of patients with obesity and diabetes who have atrial fibrillation: the potential mediating influence of epicardial adipose tissue

**DOI:** 10.1186/s12933-019-0927-9

**Published:** 2019-09-24

**Authors:** Milton Packer

**Affiliations:** 1 Baylor Heart and Vascular Institute, Baylor University Medical Center, 621 N. Hall Street, Dallas, TX 75226 USA; 2 Imperial College, London, UK

**Keywords:** Obesity, Diabetes, Atrial fibrillation, Epicardial adipose tissue

## Abstract

Both obesity and type 2 diabetes are important risk factors for atrial fibrillation (AF), possibly because they both cause an expansion of epicardial adipose tissue, which is the source of proinflammatory adipocytokines that can lead to microvascular dysfunction and fibrosis of the underlying myocardium. If the derangement of epicardial fat adjoins the left atrium, the result is an atrial myopathy, which is clinically manifest as AF. In patients with AF, there is a close relationship between epicardial fat volume and the severity of electrophysiological abnormalities in the adjacent myocardial tissues, and epicardial fat mass predicts AF in the general population. The expansion of epicardial adipose tissue in obesity and type 2 diabetes may also affect the left ventricle, impairing its distensibility and leading to heart failure with a preserved ejection fraction (HFpEF). Patients with obesity or type 2 diabetes with AF often have HFpEF, but the diagnosis may be missed, if dyspnea is attributed to increased body mass or to the arrhythmia. The expected response to the treatment for obesity, diabetes or AF may be influenced by their effects on epicardial inflammation and the underlying atrial and ventricular myopathy. Bariatric surgery and metformin reduce epicardial fat mass and ameliorate AF, whereas insulin promotes adipogenesis and cardiac fibrosis, and its use is accompanied by an increased risk of AF. Rate control strategies for AF may impair exercise tolerance, because they allow for greater time for ventricular filling in patients who cannot tolerate volume loading because of cardiac fibrosis and HFpEF. At the same time, both obesity and diabetes decrease the expected success rate of rhythm control strategies for AF (e.g., electrical cardioversion or catheter ablation), because increased epicardial adipose tissue volumes and cardiac fibrosis are important determinants of AF recurrence following these procedures.

Both obesity and type 2 diabetes are important risk factors for the development of atrial fibrillation (AF). Although hypertension has long been the primary determinant of AF in the general community, obesity represents the second highest population-attributable risk for AF, and its importance is growing [1]. An increase in body mass contributes to the development of AF in 20% of patients with AF, and short-term weight gain elevates the risk of AF by 40% over a follow-up of 5 years [1, 2]. At the same time, diabetes also contributes significantly to the development of AF; the severity of hyperglycemia predicts the incidence of AF [3].

## Role of obesity- and diabetes-driven epicardial adipose tissue expansion in mediating the development of atrial fibrillation

**Fig. 1:**
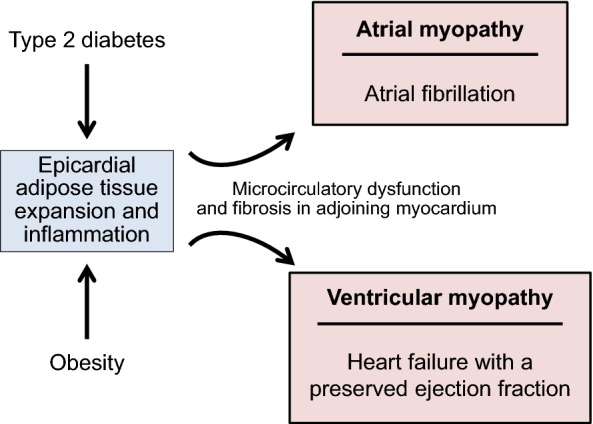
Mechanisms by which metabolic disorders may simultaneously cause atrial and ventricular myopathy, leading to atrial fibrillation and to heart failure with a preserved ejection fraction

What mechanisms drive the development of AF in these two common metabolic disorders? Both obesity and type 2 diabetes are accompanied by an expansion and biological transformation of epicardial adipose tissue [4–7], which can be the source of proinflammatory mediators that are capable of causing microvascular dysfunction and fibrosis of the underlying myocardium [8–10]. If the derangement of epicardial fat adjoins the left atrium, the result is electroanatomical remodeling leading to an atrial myopathy (Fig. 1) [11]. In patients with AF and other cardiovascular disorders, there is a close relationship between the volume and inflammatory state of epicardial fat, the presence of atrial fibrosis, and the severity of electrophysiological abnormalities in the adjacent myocardial tissues [5, 12–14]. Epicardial fat mass predicts the incidence of AF in the general population [15]; it increases as AF progresses from a paroxysmal to a persistent arrhythmia [16]; and it identifies patients at risk of major adverse cardiovascular events [17]. The powerful link between obesity and the risk of AF in epidemiological studies is entirely explained by the underlying atrial myopathy [18].

Importantly, the expansion and inflammation of epicardial adipose tissue in obesity and type 2 diabetes affects not only the atria, but also the ventricles [11]. The derangements of epicardial fat can lead to inflammation, microcirculatory dysfunction and fibrosis in the adjoining myocardium, impairing the distensibility of the left ventricle (LV) and restraining its ability to tolerate volume (Fig. 1) [11, 19, 20]. LV filling pressure rises, causing exertional dyspnea and leading to heart failure with a preserved ejection fraction (HFpEF) [19]. Incident AF increases the risk of a subsequent diagnosis of heart failure, particularly HFpEF [21, 22]. Even when heart failure has not been formally diagnosed, many patients with AF (particularly with exercise intolerance) have increased LV filling pressure at rest or during exercise on echocardiography or by cardiac catheterization [23–25]. Therefore, patients with obesity or type 2 diabetes with AF often have underlying latent HFpEF, but the diagnosis is frequently not made, because dyspnea is often attributed to increased body mass or to the arrhythmia.

## Disease–treatment interactions in patients with obesity or type 2 diabetes who have atrial fibrillation

How should patients with obesity or diabetes who have AF be managed? Physicians could (1) treat the causal metabolic disorder or (2) directly address the arrhythmia, either with rate- or rhythm-control strategies. However, the expected response to these interventions may be influenced by epicardial adipose tissue inflammation and by the underlying atrial and ventricular myopathy.

### Influence of weight loss and antihyperglycemic drugs on AF

Epicardial fat is relatively resistant to weight loss regimens [26]; thus, the modest weight loss that is typically seen with caloric restriction has minimal effect on epicardial adipose tissue [27] and exerts little benefit on AF [28]. In contrast, marked weight loss (e.g., with bariatric surgery) can decrease both the mass and inflammation of epicardial fat [29, 30]. In both observational studies and randomized controlled trials, striking degrees of weight loss can reduce the burden of AF or restore sinus rhythm in patients with established AF [31, 32]. Interestingly, this degree of weight loss is also paralleled by an amelioration of the diastolic filling abnormalities typically seen in HFpEF [33].

Additionally, in patients with type 2 diabetes, the effects of antihyperglycemic drugs on AF may parallel their actions on epicardial adipose tissue. Insulin promotes adipogenesis and cardiac fibrosis [34, 35] (exacerbating the atrial myopathy) and exerts antinatriuretic effects (increasing atrial wall stress) [36]. Also, by promoting episodic hypoglycemia, insulin may activate the sympathetic nervous system and enhance arrhythmogenesis. Accordingly, insulin use is accompanied by an increased risk of AF [37, 38]. In contrast, metformin exerts anti-inflammatory effects on adipose tissue and decreases the release of proinflammatory adipokines from the epicardium [39, 40], and its use has been accompanied by a decreased risk of AF [41]. In addition, by promoting PPAR-γ signaling, pioglitazone can reverse the dysfunctional state of epicardial fat [42, 43], thereby attenuating atrial inflammation and fibrosis. Pioglitazone ameliorates AF in experimental models [44], and use of the drug has been associated with a lower risk of new-onset or recurrent AF in observational studies in the clinical setting [45, 46]. However, thiazolidinediones did not reduce AF events in two randomized controlled trials of patients with insulin resistance or type 2 diabetes [47], possibly because PPAR-γ agonism promotes sodium retention and increases cardiac volumes [48]. The resulting atrial distension could negate any benefits on AF that might be expected from the action of these drugs to reduce epicardial adipose inflammation.

### Rate- and rhythm-control strategies for AF in obesity and diabetes

Even though patients with obesity and type 2 diabetes are at high risk of undiagnosed HFpEF, physicians will often ascribe complaints of dyspnea to the presence of AF, and thus, treatments are likely to be directed towards control of AF. However, efforts at both rate and rhythm control are often unsuccessful and carry important risks, if patients have a metabolic disorder or underlying HFpEF.

#### Rate control strategies

The intent of rate control in AF is to prevent tachyarrhythmia-related cardiac injury. However, in most patients with heart failure and AF, a rapid ventricular rate does not have adverse functional or prognostic significance. When compared with patients with faster heart rates, patients with greater rate control do not have improved long-term outcomes [49]. Furthermore, if there is underlying HFpEF, heart rate slowing can impair exercise tolerance, presumably because it allows greater time for ventricular filling in patients who cannot tolerate volume loading because of cardiac fibrosis [50]. The use of atrioventricular nodal blocking drugs (e.g., digoxin, amiodarone and dronedarone) has been associated with an increased risk of death in patients with AF [51, 52]. Furthermore, although beta-blockers reduce morbidity and mortality in patients with a reduced ejection fraction in sinus rhythm, they do not exert these benefits in those with AF, particularly if the ejection fraction is preserved [53]. Fibrosis-related conduction system disease may increase the risk of serious bradyarrhythmias if patients are prescribed rate-control agents [54]. These experiences raises important doubts about the value of intensive rate control of AF in patients with obesity or diabetes, who are likely to have an underlying inflammatory myopathy.

#### Rhythm control strategies

Given these challenges, physicians frequently turn to rhythm control strategies for AF, i.e., electrical or chemical cardioversion or catheter ablation. However, both obesity and type 2 diabetes decrease the success rate (i.e., maintenance of sinus rhythm) following electrical cardioversion [55, 56], presumably because increased epicardial adipose tissue volume is a major determinant of AF recurrence following the procedure [57]. Furthermore, the post-cardioversion administration of anti-arrhythmic drugs carries an important risk of proarrhythmia and worsening heart failure, particularly in patients who have an underlying atrial or ventricular myopathy [52].

Catheter ablation may be used to abolish AF in patients with obesity or type 2 diabetes. However, the left atrium and LV in these individuals is typically affected with extensive fibrosis [58], especially if they have long-standing AF [59]. Unfortunately, patients with AF who have myocardial fibrosis are unlikely to maintain sinus rhythm following ablation [60, 61]—especially if epicardial adipose tissue volume is increased [62, 63]—thus explaining the high rate of AF recurrence in patients with obesity or type 2 diabetes [64–66]. Marked weight loss produced by bariatric surgery (which reduces epicardial fat volume) can improve the success of ablation procedures [67]. However, if epicardial adiposity persists, the presence of fibrosis may attenuate any benefit that abolition of AF might otherwise have on LV structure and function. In the only trial that has reported favorable effects of catheter ablation on LV ejection fraction using magnetic resonance imaging, the observed benefit was confined to those without preprocedural myocardial fibrosis [68]. More worrisome, if a patient with AF also has an underlying atrial myopathy as a result of epicardial inflammation caused by obesity or diabetes, ablation may add to the pre-existing fibrotic burden of the left atrium, further compromising chamber capacitance and leading to post-procedural increases in pulmonary venous pressures and worsening heart failure, particularly in those with underlying HFpEF [69, 70].

## Conclusions

Patients with obesity or type 2 diabetes are at markedly increased risk of AF. The management of the metabolic disorder can influence the course of AF, and conversely, efforts to treat AF (with rate or rhythm control) may have limited efficacy in patients with these coexistent conditions. It is hypothesized that these complex interactions are mediated by an expansion of epicardial adipose tissue, which not only drives the development of AF, but whose biology may also be influenced by the management of the underlying metabolic diseases. Longitudinal studies using magnetic resonance imaging to quantify epicardial fat and cardiac fibrosis are poised to confirm or refute this hypothesis.

## Data Availability

There are no new data presented; this is a commentary

## Abbreviations

AF: atrial fibrillation
HFpEF: heart failure with a preserved ejection fraction
PPAR-γ: peroxisome proliferator-activated receptor-gamma
